# Parallel STEPS: Large Scale Stochastic Spatial Reaction-Diffusion Simulation with High Performance Computers

**DOI:** 10.3389/fninf.2017.00013

**Published:** 2017-02-10

**Authors:** Weiliang Chen, Erik De Schutter

**Affiliations:** Computational Neuroscience Unit, Okinawa Institute of Science and Technology Graduate UniversityOkinawa, Japan

**Keywords:** STEPS, parallel simulation, stochastic, spatial reaction-diffusion, HPC

## Abstract

Stochastic, spatial reaction-diffusion simulations have been widely used in systems biology and computational neuroscience. However, the increasing scale and complexity of models and morphologies have exceeded the capacity of any serial implementation. This led to the development of parallel solutions that benefit from the boost in performance of modern supercomputers. In this paper, we describe an MPI-based, parallel operator-splitting implementation for stochastic spatial reaction-diffusion simulations with irregular tetrahedral meshes. The performance of our implementation is first examined and analyzed with simulations of a simple model. We then demonstrate its application to real-world research by simulating the reaction-diffusion components of a published calcium burst model in both Purkinje neuron sub-branch and full dendrite morphologies. Simulation results indicate that our implementation is capable of achieving super-linear speedup for balanced loading simulations with reasonable molecule density and mesh quality. In the best scenario, a parallel simulation with 2,000 processes runs more than 3,600 times faster than its serial SSA counterpart, and achieves more than 20-fold speedup relative to parallel simulation with 100 processes. In a more realistic scenario with dynamic calcium influx and data recording, the parallel simulation with 1,000 processes and no load balancing is still 500 times faster than the conventional serial SSA simulation.

## Introduction

Recent research in systems biology and computational neuroscience, such as the study of Purkinje cell calcium dynamics (Anwar et al., 2014), has significantly boosted the development of spatial stochastic reaction-diffusion simulators. These simulators can be separated into two major categories, voxel-based and particle-based. Voxel-based simulators, such as STEPS (Hepburn et al., 2012), URDME (Drawert et al., 2012), MesoRD (Hattne et al., 2005), and NeuroRD (Oliveira et al., 2010), divide the geometry into small voxels where different spatial variants of the Gillespie Stochastic Simulation Algorithm (Gillespie SSA) (Gillespie, 1976) are applied. Particle-based simulators, for example, Smoldyn (Andrews and Bray, 2004) and MCell (Kerr et al., 2008), track the Brownian motion of individual molecules, and simulate molecular reactions caused by collisions. Although greatly successful, both voxel-based and particle-based approaches are computationally expensive. Particle-based simulators suffer from the requirement of tracking the position and movement of every molecule in the system. While tracking individual molecules is not required for voxel-based simulators, the exact solution of Gillespie SSA is highly sequential and inefficient for large-scale simulation due to the massive amount of SSA events (Dematté and Mazza, 2008).

There is a major need for more efficient stochastic spatial reaction-diffusion simulation of large-scale systems. Over the years several efforts have achieved considerable success, both in algorithm development and software implementation, but increasing simulation scale and complexity have significantly exceeded the gains in speed.

Since the introduction of the original Gillespie SSA, performance of voxel-based simulators has been substantially improved thanks to new algorithms and data structures. Giving *N* as the number of possible kinetic events (reactions and diffusions) in the system, the computational complexity of a single SSA iteration has been reduced from O(*N*) with the Direct method (Gillespie, 1976), to O(log(*N*)) with Gibson and Bruck's modification (Gibson and Bruck, 2000), to O(1) with the composition and rejection SSA (Fricke and Schnakenberg, 1991; Slepoy et al., 2008). Approximate solutions for well-stirred systems, such as the well-known tau-leaping method (Gillespie, 2001) can also be applied to the spatial domain (Marquez-Lago and Burrage, 2007; Koh and Blackwell, 2011), providing further speedup with controllable errors. It is clear, however, that the performance of a serial simulator is restricted by the clock speed of a single computing core, while multi-core CPU platforms have become mainstream.

One possible way to bypass the clock speed limitation is parallelization, but development of an efficient and scalable parallel solution has proven challenging. An optimistic Parallel Discrete Event Simulation (PDES) solution has been applied to the exact Gillespie SSA, achieving a maximum 8x speedup with a 12-core cluster (Dematté and Mazza, 2008). This approach has been further investigated and tested with different synchronization algorithms available for PDES systems (Wang et al., 2009), such as Time Warp (TW), Breathing Time Bucket (BTB) and Breathing Time Warp (BTW). Their results indicate that while considerable speedup can be achieved, for example 5x speedup with 8 cores using the BTW method, speed decays rapidly once inter-node communication is involved, due to significant network latency. Another optimization attempt using the PDES solution with thread-based implementation achieved a 9x acceleration with 32 processing threads (Lin et al., 2015). All the foregoing studies show scalability limitations due to the dramatic increase in rollbacks triggered by conflicting diffusion events between partitions, even with support from well-developed PDES algorithms.

Parallelization of approximate SSA methods has also been investigated. D'Agostino et al. (2014) introduced a parallel spatial tau-leaping solution with both Message Passing Interface (MPI)-based and Graphics Processing Unit (GPU)-based implementations, achieving a 20-fold acceleration with 32 CPU cores, and about 50x on a 192-core GTX-Titan. Two variants of the operator-splitting approach, originating from the serial Gillespie Multi-Particle (GMP) method (Rodríguez et al., 2006), have been independently employed by Roberts (Roberts et al., 2013) and Vigelius (Vigelius et al., 2011). Both GPU implementations achieve more than 100-fold speedup compared to the CPU-based serial SSA implementations. It is worth noting that the above-mentioned parallel solutions divide simulated geometries into sub-volumes using cubic mesh grids, which may not accurately represent realistic morphologies (Hepburn et al., 2012).

Several studies of parallel particle-based implementations have been reported. Balls et al. (2004) demonstrated their early attempt at parallel MCell implementation under the KeLP infrastructure (Fink et al., 1998) with a 64-core cluster. Two GPU-based parallel implementations of Smoldyn have also been reported (Gladkov et al., 2011; Dematté, 2012); both show 100~200-fold speedup gains compared to the CPU-based serial Smoldyn implementation.

Here we introduce an MPI-based parallel implementation of the STochastic Engine for Pathway Simulation (STEPS) (Hepburn et al., 2012). STEPS is a GNU-licensed, stochastic spatial reaction-diffusion simulator implemented in C++ with a Python user interface. The main solver of serial STEPS simulates reaction and diffusion events by applying a spatial extension of the composition and rejection SSA (Slepoy et al., 2008) to sub-volumes of unstructured tetrahedral meshes. Our parallel implementation aims to provide an efficient and scalable solution that can utilize state-of-the-art supercomputers to simulate large scale stochastic reaction-diffusion models with complex morphologies. In Section Methods we explain the main algorithm and details essential to our implementation. In Section Results, we then showcase two examples, from a simple model to a complex real-world research model, and analyze the performance of our implementation with their results. Finally, we discuss possible future developments of parallel STEPS in Section Discussion and Future Directions.

## Methods

We choose the MPI protocol for CPU clusters as the development environment of our parallel implementation, since it is currently the best-supported parallel environment in academic research. Modern clusters allow us to explore the scalability of our implementation with a massive number of computing nodes, and provide insights for further optimization for super-large-scale simulations. The MPI-based implementation also serves as the foundation of future implementations with other parallel protocols and hardware, such as GPU and Intel Xeon Phi clusters.

Previous attempts (Dematté and Mazza, 2008; Wang et al., 2009; Lin et al., 2015) to parallelize the exact Gillespie SSA have shown that system rollbacks triggered by straggler cross-process diffusion events can negate any performance gained from parallelization. The issue is further exacerbated for MPI-based implementations due to significant network latency. To take full advantage of parallelization, it is important to relax exact time dependency of diffusion events and to take an approximate, time-window approach that minimizes data communication and eliminates system rollbacks. Inspired by the GMP method, we developed a tetrahedral-based operator-splitting algorithm as the fundamental algorithm of our parallel implementation. The serial implementation of this algorithm and its accuracy have been discussed previously (Hepburn et al., 2016). Here we discuss implementation details of the parallel version.

### Initialization of a parallel STEPS simulation

To initialize a parallel STEPS simulation, the user is required to provide the biochemical model and geometry to the parallel solver. For user convenience, our parallel implementation accepts the same biochemical model and geometry data used as inputs in the serial SSA solver. In addition, mesh partitioning information is required so that tetrahedrons can be distributed and simulated. Partitioning information is a simple list that can be generated automatically using the grid-based partitioning solution provided in the STEPS utility module, or more sophisticated, third-party partitioning applications, such as Metis (Coupez et al., 2000). The STEPS utility module currently provides necessary support functions for format conversions between STEPS and Metis files.

Assuming that a set of tetrahedrons is hosted by an MPI process *p*, {*tet*|*tet* is hosted by *p*}, parallel STEPS first creates a standard Gillespie SSA system for all reactions in each hosted tetrahedron. This includes the population state of all molecule species and propensities of reactions. For each reaction *R*_*tet*,*p*_, it also creates an update dependency list deps(*R*_*tet,p*_), that is, a list of reactions and diffusions that require an update if *R*_*tet*,*p*_ is chosen and applied by the SSA. Since a reaction only affects molecule states and propensities of reactions and diffusions within its own tetrahedron, the above information can be stored locally in *p*. The localized storage of SSA and dependency information significantly reduces memory consumption for each process compared to a serial SSA implementation, which is crucial to simulator performance. We will further address its importance with simulation results in section Results.

The simulation also stores the set of hosted diffusion processes {*D*_*tet*,*p*_|*D*_*tet*,*p*_ is in *tet* hosted by *p*} and the dependency list deps(*D*_*tet*, *p*_) for each diffusion *D*_*tet*,*p*_. In addition, if a tetrahedron *tet* is a boundary tetrahedron of *p*, in other words, the molecule state of *tet* is affected by diffusions in tetrahedrons hosted by other MPI processes rather than *p*, a species update dependency list for every diffusive species *S*_*tet*,*p*_ in *tet* is also created. The species update dependency list, deps(*S*_*tet,p*_), is defined as the list of reactions and diffusions that are hosted by *p*, and that require an update if the count of *S*_*tet*,*p*_ is modified by cross-process diffusion. The species dependency list allows each MPI process to update hosted reactions and diffusions independently after receiving molecule change information from other processes, thus reducing the need for cross-process communication.

Furthermore, a suitable diffusion time window is determined according to the biochemical model and geometry being simulated (Hepburn et al., 2016). Given *d*_*S,tet*_ as the local diffusion rate for diffusive species *S* in tetrahedron *tet*, each process *p* computes a local minimal time window $\tau_{p}=\text{min}\frac{1}{d_{S,tet}}$, over all diffusive species in every hosted tetrahedron. Collective communication is then performed to determine the global minimum, τ = min(τ_*p*_), which is set as the diffusion time window for every process in the simulation. Note that τ is completely determined by the biochemical model and geometry, and remains constant regardless of changes in the molecule population. Therefore, continual updates of τ are not required during the simulation.

The final step is to initialize the molecule population state of the simulation, which can be done using various API functions provided in parallel STEPS. Once this is completed, the simulation is ready to enter the runtime main loop described below.

### Runtime main loop

The runtime main loop for each MPI process is shown in Algorithm 1 in Supplementary Material. When a process is asked to execute the simulation from time *t* to *t*_*end*_, a remote change buffer for cross-process data communication is created for each of the neighboring processes of *p*. Details of the buffer will be discussed later.

The entire runtime [*t, t*_*end*_] is divided into iterations of the constant time window τ, the value of which is computed during initialization. At the start of every time window, each process first executes the Reaction SSA operator for the period of τ. The mean number of a molecule species *S* present in a tetrahedron *tet* during τ is used to determine the number of *S* to be distributed among neighbors of *tet*. Therefore, in addition to the standard exact SSA routine, the process also updates time and occupancy for each reactant and product species (Hepburn et al., 2016).

The parallel solver treats diffusion events differently, based on ownerships of tetrahedrons involved. If both source and destination tetrahedrons of a diffusion event are in a single process, diffusion is applied directly. If a diffusion is cross-process, that is, the source tetrahedron and destination tetrahedron are hosted by different processes, the change to the source tetrahedron is applied directly, while the change to the destination tetrahedron is registered to the corresponding remote change buffer. Once all diffusion events are applied or registered, the buffers are sent to associated remote processes via non-blocking communication, where molecule changes in destination tetrahedrons are applied.

**Figure 1:**
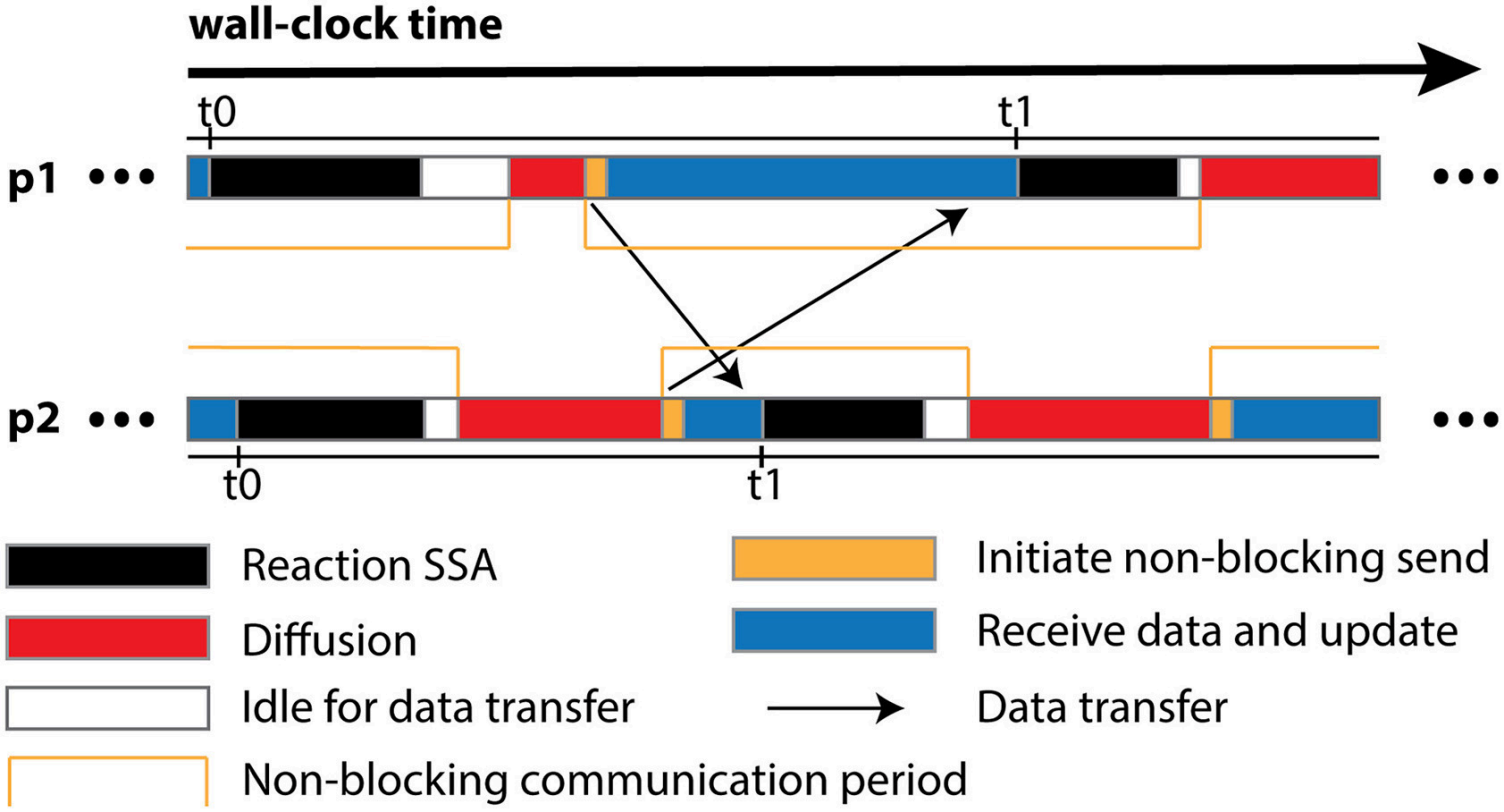
**Schematic illustration of different runtime stages of two processes, ***p***_**1**_ and ***p***_**2**_, assuming that ***p***_**1**_ is the only neighboring process of ***p***_**2**_**. Once *p*_2_ receives and applies the remote change buffer from *p*_1_ for iteration *t*_0_, it can immediately start the reaction SSA computation for iteration *t*_1_, without waiting for *p*_1_ to complete iteration *t*_0_. Due to non-blocking communication mechanism, the actual data transfer may take place any time within the communication period. Data communication between *p*_1_ and its neighboring processes except *p*_2_ is skipped for simplification.

The algorithm is designed for optimal operation in a parallel environment. Most of its operations can be performed independently without network communication. In fact, the only data communication required is the transfer of remote change buffers between neighboring processes. This has two important implications. First and foremost, the communication is strictly regional, meaning that each process only communicates to a small subset of processes with which it shares geometric boundaries, regardless of the overall scale of the simulation. Secondly, thanks to the non-blocking communication, each process can start the Reaction SSA Operator for the next iteration *t*_1_, as soon as it receives remote change buffers for the current iteration *t*_0_ from all neighboring processes and applies those changes (Figure 1). Therefore, data communication can be hidden behind computation, which helps to reduce the impact of network latency.

**Figure 2:**
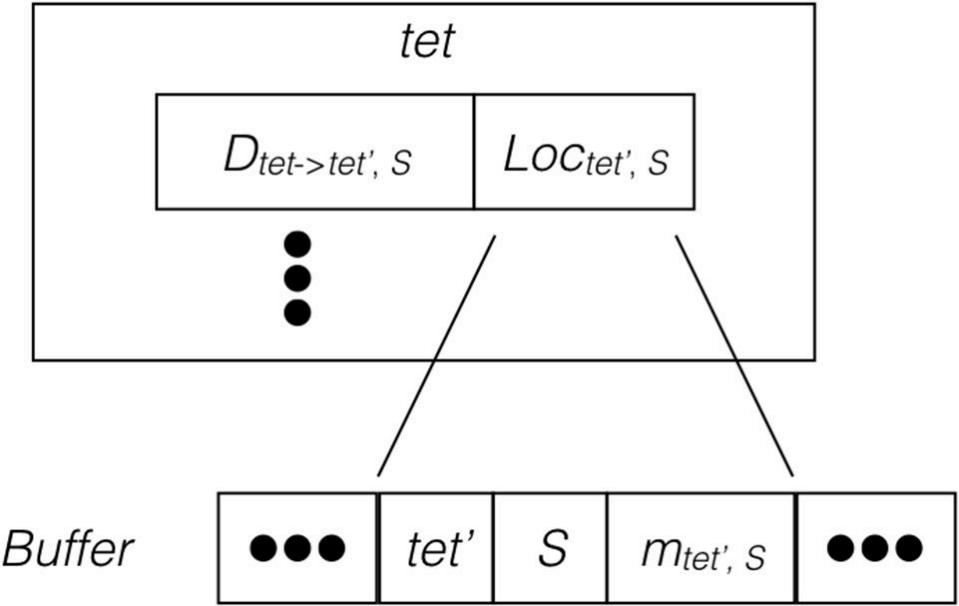
**Schematic illustration of the remote change buffer data structure**. For every cross-process diffusion event taking place in *tet*, it first compares its destination tetrahedron and species information with the entry data stored at *Loc*_*tet*′,*S*_ of the remote change buffer. If the match is successful, the accumulated change of this entry is increased, otherwise a new entry is appended to the buffer.

Since the remote change buffer holds the only data being transferred across the network, it is important to limit its size so that communication time can be reduced. Furthermore, an efficient registering method is also required since all molecule changes applied to remotely hosted tetrahedrons need to be recorded. Algorithm 2 in Supplementary Material and Figure 2 illustrate the procedure and data structure for the registration. Instead of recording every cross-process diffusion event, the remote change buffer records the accumulated change of a molecule species in a remotely hosted tetrahedron. Thus, the size of the remote change buffer has an upper bound corresponding to the number of remotely-hosted neighboring tetrahedrons and the number of diffusive species within those tetrahedrons. The lower bound is zero if no cross-process diffusion event occurs during an iteration. The remote change buffer is a vector that stores entries of molecule changes sequentially. Each entry consists of three elements, the destination tetrahedron *tet*′, the diffusive species *S*, as well as its accumulated change *m*_*tet*′,*S*_. All elements are represented by integers. For every possible cross-process diffusion event, *D*_*tet*→*tet*′,*S*_, the host process stores a location marker *Loc*_*tet*′,*S*_, that indicates where the corresponding entry is previously stored in the buffer. When a cross-process diffusion event occurs, the host process of the source tetrahedron first compares the destination tetrahedron and information about the diffusive species to the entry data stored at the marked location in the buffer. If the data match the record, the accumulated change of this entry is increased according to the diffusion event. Each buffer is cleared after its content has been sent to corresponding processes, thus a mismatch of entry information indicates that a reset has taken place since the previous registration of the same diffusion event, in which case a new entry is appended to the end of the buffer and the location of this entry is stored at the location marker for future reference. Both accessing entry data and appending new entries have constant complexity with C++ Standard Template Library (STL) vectors, providing an efficient solution for registering remote molecule changes.

## Results

Because the accuracy of the solution method has been examined previously (Hepburn et al., 2016), here we mainly focus on the performance and scalability of our implementation. The implementation passed all validations, and simulation results were checked with serial SSA solutions. It is worth mentioning that the diffusion time window approximation unavoidably introduces small errors into simulations, as discussed in the publication above. Simulations reported in this paper were run on OIST's high performance cluster, “Sango.” Each computing node on Sango has two 12-core 2.5 GHz Intel Xeon E5-2680v3 processors, sharing 128 GiB of system memory. All nodes are interconnected using 56 Gbit/s InfiniBand FDR. In total, Sango comprises 10,224 computing cores and 60.75 TiB of main memory. Due to the sharing policy, only a limited number of cores could be used for our tests. Cluster conditions were different for each test and in some cases, computing cores were scattered across the entire cluster. Unfortunately, cluster conditions may affect simulation performance. To measure this impact and to understand how our implementation performs under real-life cluster restrictions, we repeated the tests multiple times, each starting at a different date and time with variable cluster conditions. For simulations with the simple model (Section Reaction-Diffusion Simulation with Simple Model and Geometry) we were able to limit the number of cores used per processor to 10. We were unable to exert the same control over large-scale simulations due to resource restriction. In all cases, hyper-threading was deactivated. Our results show that the standard deviations in wall-clock time amount to ~1% of the mean results; therefore, only mean results are reported.

Simulation performance was measured by both speedup and efficiency. Each simulation was run for a predefined period, and the wall-clock time was recorded. Given a problem with fixed size, the average wall-clock time for a set of repeated simulations to solve this problem is denoted as *T*_*p*_, where *p* is the number of MPI processes used in each simulation. The speedup of a parallel simulation with *p* processes relative to one with *q* processes is defined as *S*_*p/q*_ = *T*_*q*_ / *T*_*p*_. Specifically, the speedup of parallel simulation with *p* processes relative to its serial SSA counterpart is defined as *S*_*p/SSA*_ = *T*_*SSA*_ / *T*_*p*_, where *T*_*SSA*_ is the wall-clock time for the same simulation run by the serial SSA solver. Note that while sharing many similarities, the parallel operator-splitting implementation and the serial SSA implementation have different algorithms, data structures as well as core routines, so there is no guarantee that *S*_1/SSA_ equals one. We further define the strong scaling efficiency of a simulation with *p* processes relative to one with *q* processes as $E_{p/q}=S_{p/q}\cdot \frac{q}{p}$. Strong scaling efficiency is used to investigate the scalability performance of parallel implementations of fixed-size problems.

Scalability of a parallel implementation can also be studied by scaling both process count and problem size of the simulation together, called the weak scaling efficiency. Given *T*_*N,p*_ as the wall-clock time of a *p*-process simulation with problem size *N*, and *T*_*kN,kp*_ as the wall-clock time of another simulation in which both problem size and the number of processes are multiplied by *k* times, we define the weak scaling efficiency as *E*_*k*_ = *T*_*N,p*_ / *T*_*kN,kp*_. We will investigate both scalability performances of our implementation in later sections.

### Reaction-diffusion simulation with simple model and geometry

We first examine simulation results of a fixed-size reaction-diffusion problem. The simulated model (Table 1) was previously used to benchmark our serial spatial SSA solver (Hepburn et al., 2012) and to test the accuracy of our serial operator-splitting solution (Hepburn et al., 2016). It consists of 10 diffusive species, each with differing diffusion coefficients and initial molecule counts, and 4 reversible reactions with various rate constants. The model was simulated in a 10 × 10 × 100μm^3^ cuboid mesh with 3363 tetrahedrons. It is worth mentioning that different partitioning approaches can affect simulation performance dramatically, as will be shown hereafter. Here we partitioned the tetrahedrons linearly based on the y and z coordinates of their barycenters (the center of mass) where the numbers of partitions of each axis for a simulation with *p* processes was arranged as [Parts_x_ = 1, Parts_y_ = 5, Parts_z_ = *p*/5]. At the beginning of each simulation, species molecules were placed uniformly into the geometry, and the simulation was run for *t*_*end*_ = 20 s, after which the wall-clock time was recorded. We started each series of simulations from *p* = 5 and progressively increased the number of processes in increments of 5 until *p* = 300. Each series was repeated 30 times to produce an average result.

**Table 1:** **Simple reaction-diffusion model**.

**Species**	**Diffusion Coefficient (μm^2^/s)**	**Initial Count**
A	100	1,000
B	90	2,000
C	80	3,000
D	70	4,000
E	60	5,000
F	50	6,000
G	40	7,000
H	30	8,000
I	20	9,000
J	10	10,000
**Reaction**	**Rate Constant**	
*A* + *B* ⇄ *C*	$k_{f}:1,000\;{\left(\mu \text{M}\cdot \text{s}\right)}^{-1}$, $k_{b}:100{\text{s}}^{-1}$	
*C* + *D* ⇄ *E*	$k_{f}:100\;{\left(\mu \text{M}\cdot \text{s}\right)}^{-1}$, $k_{b}:10{\text{s}}^{-1}$	
*F* + *G* ⇄ *H*	$k_{f}:10\;{\left(\mu \text{M}\cdot \text{s}\right)}^{-1}$, $k_{b}:1{\text{s}}^{-1}$	
*H* + *I* ⇄ *J*	$k_{f}:1\;{\left(\mu \text{M}\cdot \text{s}\right)}^{-1}$, $k_{b}:1{\text{s}}^{-1}$	

**Figure 3:**
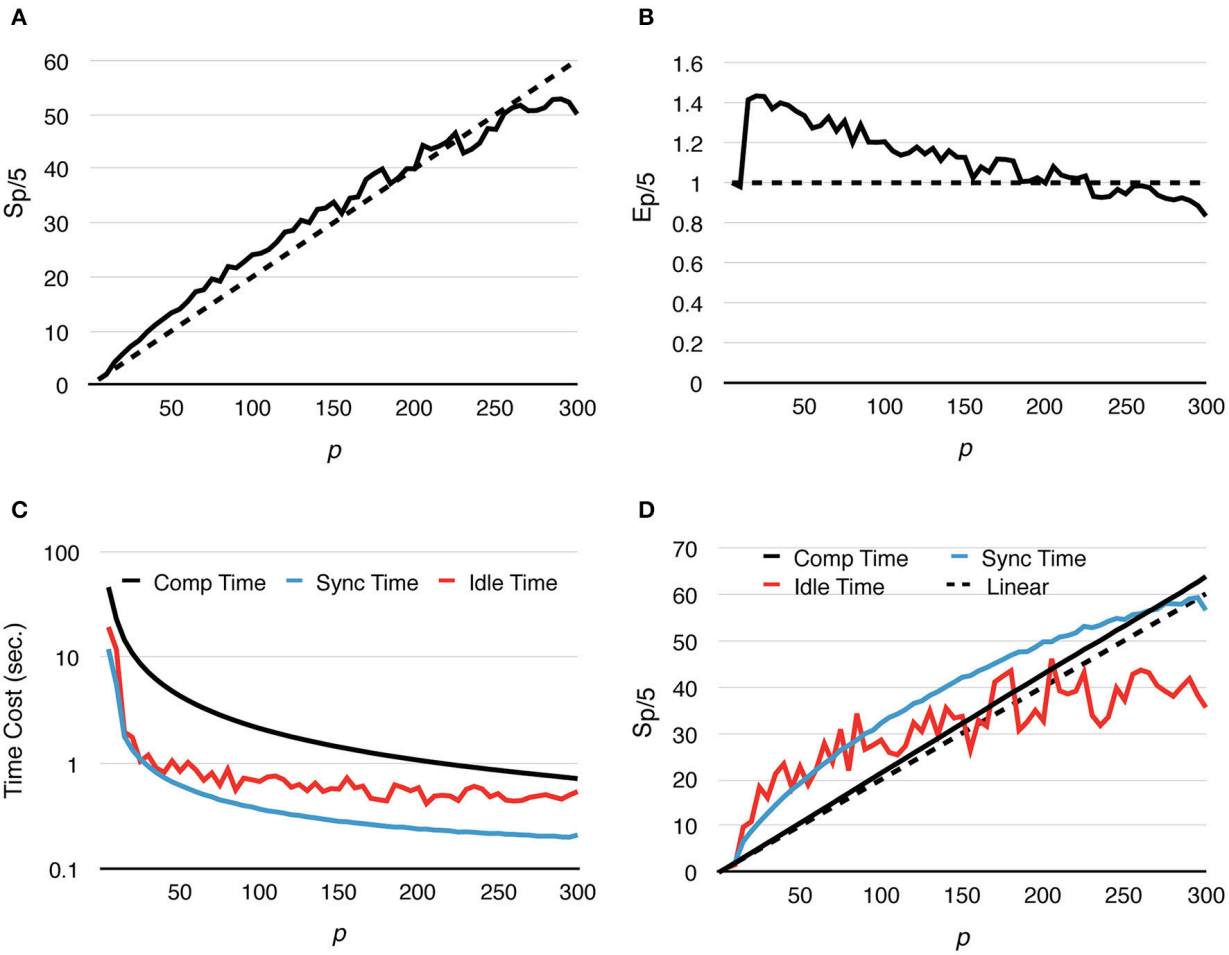
**Strong scaling performance of parallel simulations with a simple model and geometry**. Each series starts from *p* = 5 and progressively increases to *p* = 300. Both speedup and efficiency are measured relative to simulations with *p* = 5. **(A)** Simulations maintain super-linear speedup until *p* ≈ 200. **(B)** In general, efficiency decreases as *p* increases, but remains above 0.8 in the worst case (*p* = 300). **(C**,**D)** *T*_*comp*_ accounted for most of the acceleration, as it is the most time-consuming segment during simulation; it maintains super-linear speedup throughout the whole series. However, as *T*_*comp*_ decreases, *T*_*idle*_ becomes a critical factor because its change is insignificant once *p* exceeds 100.

Speedup and strong scaling efficiency are reported relative to the simulation result with 5 processes, in other words, *S*_*p*/5_ and *E*_*p*/5_. By increasing the number of processes, simulation performance of the fixed-size problem improves dramatically. In fact, the simulation maintains super-linear speedup until *p* ≈ 250 (Figure 3A). While efficiency decreases in general, it remains above 0.8 with *p* = 300 (Figure 3B), where on average each process hosts approximately 10 tetrahedrons.

In addition to the overall wall-clock time, we also recorded the time cost of each algorithm segment in order to analyze the behavior of the implementation. The total time cost for the simulation *T*_*total*_ is divided into three portions. The computation time *T*_*comp*_ includes the time cost for the reaction SSA and the cost of diffusion operations within the process (corresponds to the Reaction SSA Operator and Diffusion Operator in Algorithm 1 in Supplementary Material, colored black and red in Figure 1). The synchronization time *T*_*sync*_ includes the time cost for receiving remote change buffers from neighboring processes, and the time cost for applying those changes (corresponds to the Cross-Process Synchronization Period in Algorithm 1 in Supplementary Material, colored yellow and blue in Figure 1). The time spent waiting for the buffer's arrival, as well as the wait time for all buffers to be sent after completion of reaction SSA, is recorded as the idle time, *T*_*idle*_ (corresponds to the Idle Period in Algorithm 1 in Supplementary Material, colored white in Figure 1). In summary,

$$\begin{array}{l} T_{total}=T_{comp}+T_{sync}+T_{idle,} \end{array}$$

A detailed look at the time cost distribution of a single series trial (Figure 3C) suggests that the majority of the speedup is contributed by *T*_*comp*_, which is consistently above the theoretical ideal (Figure 3D), thanks to the improved memory caching performance caused by distributed storage of SSA and to update dependency information mentioned above. The result shows that *T*_*sync*_ also decreases significantly as the number of processes increases; however, as the number of boundary tetrahedrons is limited in the simulations, *T*_*sync*_ contributes the least to overall time consumption (Figure 3C). Another important finding is that the change of *T*_*idle*_ becomes insignificant when *p* > 100. Since *T*_*comp*_ and *T*_*sync*_ decrease as *p* increases, *T*_*idle*_ becomes progressively more critical in determining simulation performance.

**Figure 4:**
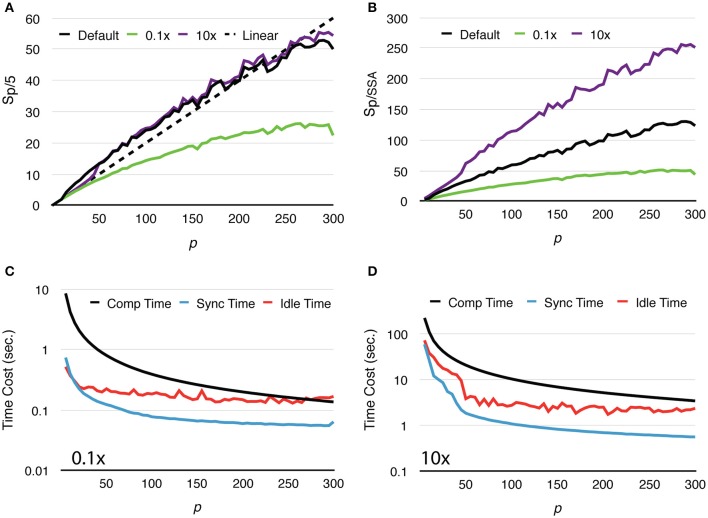
**Strong scaling performance of simulations with different molecule density. (A)** Speedups relative to simulations with *p* = 5. Simulations with low molecule density (0.1x) achieve smaller speedups compared to the default and high density (10x) cases. **(B)** In general, simulation with higher molecule density and larger scale of parallelization achieves higher speedup relative to its serial SSA counterpart. **(C)** In the 0.1x cases, *T*_*comp*_ rapidly decreases and eventually drops below *T*_*idle*_; thus, the overall speedup is less significant. **(D)** In the 10x cases, *T*_*comp*_ remains above *T*_*idle*_, therefore its contribution to speedup is significant throughout the series.

To further study how molecule density affects simulation performance, we repeated the above test with two new settings, one reduces the initial count of each molecular species by 10x, and the other increases molecule counts by 10x (Figure 4A). We named these tests “Default,” “0.1x” and “10x,” respectively. Speedups relative to the serial SSA counterparts *S*_*p*/*SSA*_ are also reported for comparison (Figure 4B). As the number of molecules in the system increases, the simulation achieves better speedup performance. This is because in the 0.1x simulations *T*_*comp*_ quickly decreases below *T*_*idle*_, and the speedup becomes less significant as *T*_*idle*_ is mostly consistent throughout the series (Figure 4C). In the 10x simulations *T*_*comp*_ maintains its domination, thus simulations achieve similar speedup ratio as the default ones (Figure 4D). This result also indicates that *S*_*p*/*SSA*_ greatly depends on molecule density. In general, parallel simulations with high molecule density and high number of processes can achieve higher speedup relative to the serial SSA counterpart (Figure 4B).

**Figure 5:**
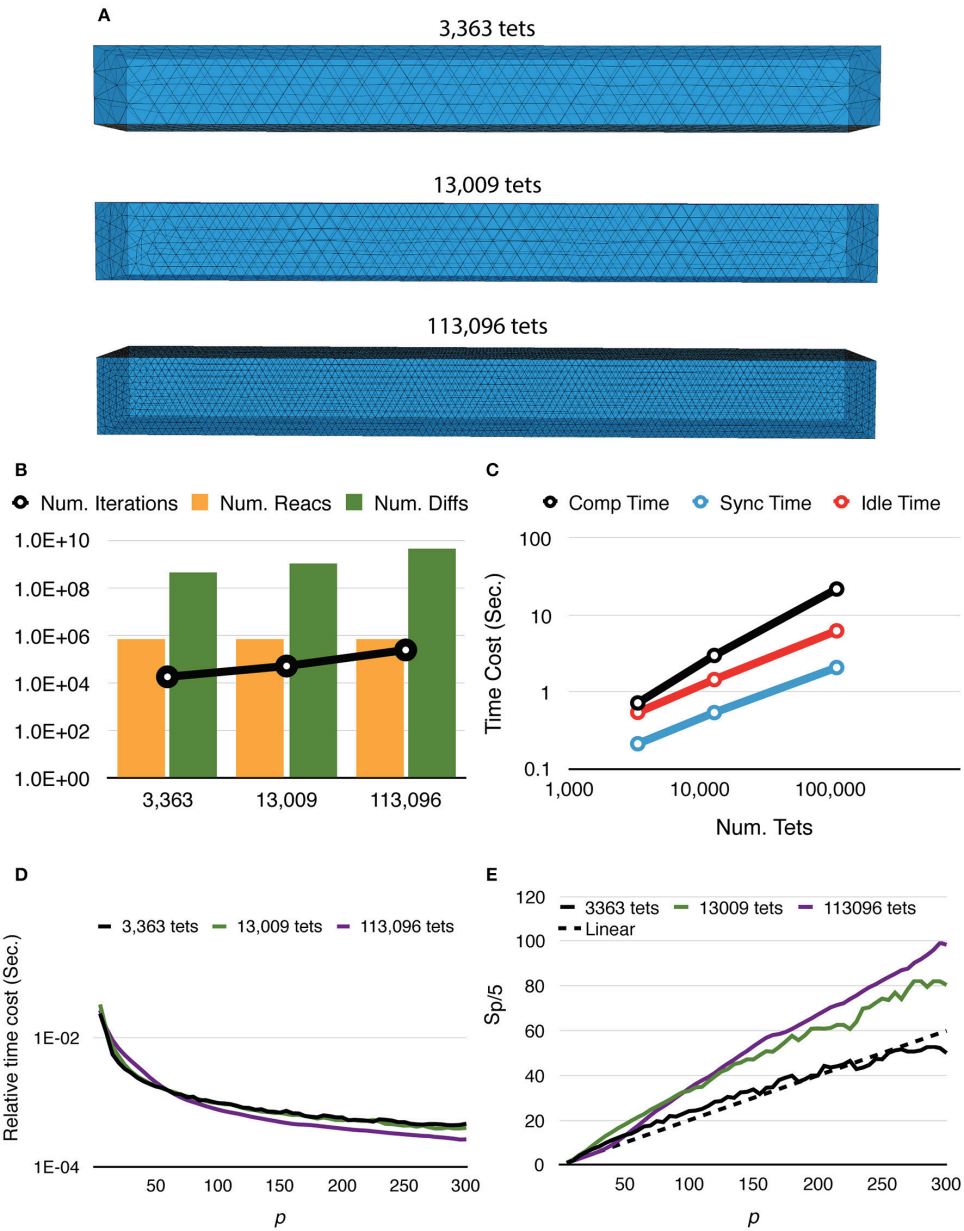
**Strong scaling performance of simulations with different mesh coarseness. (A)** Meshes with the same geometry and dimensions, but different numbers of tetrahedrons are simulated. **(B)** While the number of reaction events remains similar across mesh coarseness, both the number of diffusion events and the number of main loop iterations increase for the finer mesh. **(C)** Time distribution of simulations with *p* = 300, all three segments increase as the number of tetrahedrons increases. **(D)** Finer mesh results in a more significant decrease of relative time cost, defined as *T*_*total*_/*n*_*tets*, improving efficiency. **(E)** Speedups relative to *T*_5_. Simulation with finer mesh achieves much higher speedup in massive parallelization, thanks to the memory caching effect.

Mesh coarseness also greatly affects simulation performance. Figure 5 shows the results of simulations with the same model, geometry, and dimensions, but different numbers of tetrahedrons within the mesh. Simulations with a finer mesh generally take longer to complete because while the number of reaction events remains similar regardless of mesh coarseness, the number of diffusion events increases with a finer mesh. The number of main loop iterations also increases for finer mesh due to the inverse relationship between the diffusion time window τ and the local diffusion rate *d*_*S,tet*_ (Figure 5B). This leads to increases of all three timing segments (Figure 5C). Nevertheless, giving *n*_*tets* as the number of tetrahedrons simulated, the relative time cost of the simulation, that is, *T*_*total*_/*n*_*tets*, decreases more significantly for a finer mesh (Figure 5D), indicating improved efficiency. It is further confirmed in Figure 5E as both 13,009 and 113,096 cases achieve dramatic relative speedups from parallelization, where the 113,096 series is the most cost-efficient with high process counts. This is because in these simulations, reaction events take place stochastically over the whole extent of the mesh with no specific “hot-spot,” due to the homogeneous distribution of molecules and similar sizes of tetrahedrons. Therefore, the average memory address distance between two consecutive reaction events in each process is determined by the size of partitions hosted by the process. This distance is essential to memory caching performance. In general, smaller hosted partitions mean shorter address distances and are more cache-friendly. The performance boost from the caching effect is particularly significant for simulations with a fine mesh because the address space frequently accessed by the main loop cannot fit in the cache completely when a small number of processes is used.

**Figure 6:**
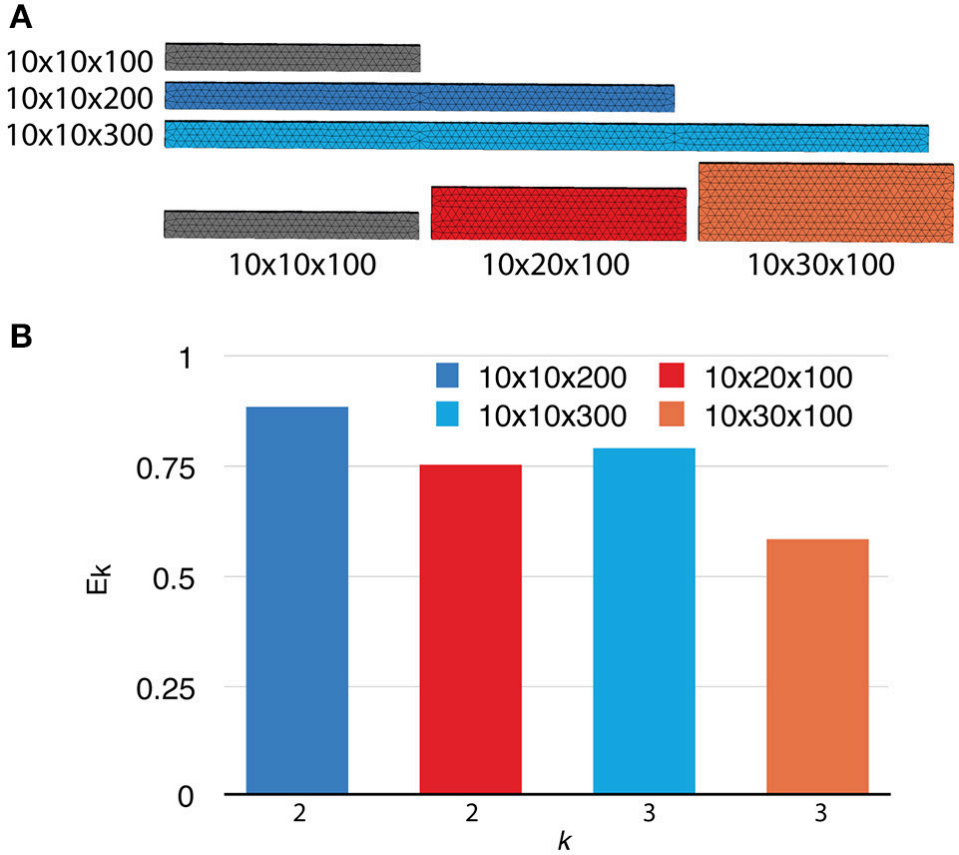
**Weak scaling performance of the implementation. (A)** The default 10 × 10 × 100μm^3^ mesh is extended along either the y or z axis as problem size increases. **(B)** Weak scaling efficiencies relative to the default case (*p* = 300).

To investigate the weak scaling efficiency of our implementation, we used the “Default” simulation with 300 processes as a baseline, and increased the problem size by duplicating the geometry along a specific axis, as well as by increasing the number of initial molecules proportionally. Table 2 gives a summary of all simulation settings. As the problem size increases, the simulation efficiency progressively deteriorates (Figure 6). While ~95% efficiency is maintained after doubling the problem size, tripling the problem size reduces the efficiency to ~80%. This is an expected outcome of the current implementation, because although the storage of reaction SSA and update dependency information are distributed, each process in the current implementation still keeps the complete information of the simulation state, including geometry and connectivity data of each tetrahedron, as well as the number of molecules within. Therefore, the memory footprint per process for storing this information increases linearly with problem size. The increased memory footprint of the simulation state widens the address distances between frequently accessed data, reducing cache prefetching performance and consequently the overall simulation efficiency. Optimizing memory footprint and memory caching for super-large-scale problems will be a main focus of our next development iteration. Our result also indicates that geometry partitioning plays an important role in determining simulation performance, as extending the mesh along the z axis gives better efficiency than extending it along the y axis, even though they have similar numbers of tetrahedrons. This can be explained by the increase of boundary tetrahedrons in the latter case. Since the number of boundary tetrahedrons determines the upper-bound of the size of remote change buffer and consequently the time for communication, reducing the number of boundary tetrahedrons is a general recommendation for geometry partitioning in parallel STEPS simulations.

**Table 2:** **Simulation settings for weak scalability study**.

**Geometry Dimensions (μm^3^)**	**Initial Count**	**Num. Processes**
10 × 10 × 100	Default	300
10 × 10 × 200	2x	600
10 × 20 × 100	2x	600
10 × 10 × 300	3x	900
10 × 30 × 100	3x	900

### Large scale reaction-diffusion simulation with real-world model and geometry

Simulations from real-world research often consist of reaction-diffusion models and geometries that are notably more complex than the ones studied above. As a preliminary example, we extracted the reaction-diffusion components of a previously published spatial stochastic calcium burst model (Anwar et al., 2013) as our test model to investigate how our implementation performs with large-scale real-world simulations. The extracted model consists of 15 molecule species, 8 of which are diffusive, as well as 22 reactions. Initial molecule concentrations, reaction rate constants and diffusion coefficients were kept the same as in the published model.

**Figure 7:**
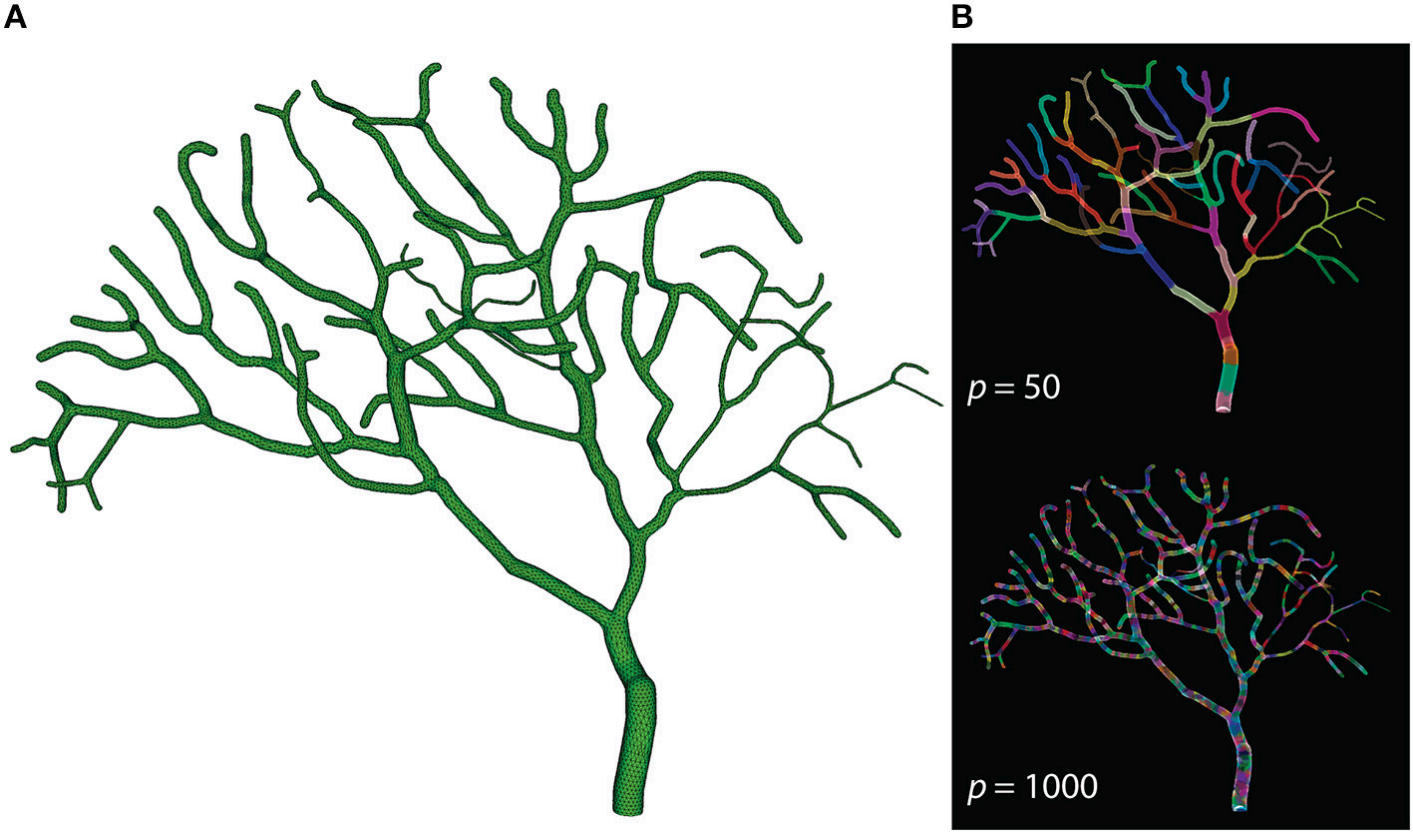
**(A)** Tetrahedral mesh of a Purkinje cell with sub-branch morphology. This mesh consists of 111,664 tetrahedrons. **(B)** Partitioning generated by Metis for *p* = 50 and *p* = 1000. Each color segment indicates a set of tetrahedrons hosted by a single process.

The Purkinje cell sub-branch morphology, published along with the model, was also used to generate a tetrahedral mesh that is suitable for parallel simulation. The newly generated mesh has 111,664 tetrahedrons, and was partitioned using Metis and STEPS supporting utilities. As discussed before, reducing boundary tetrahedrons is the general partitioning strategy for parallel STEPS simulations. This is particularly important for simulations with a tree-like morphology because a grid-based partitioning approach used for the previous models cannot capture and utilize spatial features of such morphology. The sub-branch mesh for our simulation is partitioned based on the connectivity of tetrahedrons. Specifically, a connectivity graph of all tetrahedrons in the mesh was presented to Metis as input. Metis then produced a partitioning profile which met the following criteria. First of all, the number of tetrahedrons in each partition was similar. Secondly, tetrahedrons in the same partition were all connected. Finally, the average degree of connections is minimal. Figure 7 shows the mesh itself as well as two partitioning profiles generated for *p* = 50 and *p* = 1000. As a preliminary test, this partitioning procedure does not account for any size differences of tetrahedrons and the influence from the biochemical model and molecule concentrations, although their impacts can be significant in practice. At present, some of these factors can be abstracted as weights between elements in Metis; however, substantial manual scripting is required and the solution is project-dependent.

To mimic the calcium concentration changes caused by voltage-gated calcium influx simulated in the published results (Anwar et al., 2013), we also extracted the region-dependent calcium influx profile from the results, which can be applied periodically to the parallel simulation. Depending on whether this profile is applied, the parallel simulation behaved differently. Without calcium influx, the majority of simulation time was spent on diffusion events of mobile buffer molecules. As these buffer molecules were homogeneously distributed within the mesh, the loading of each process was relatively balanced throughout the simulation. When calcium influx was applied and constantly updated during the simulation, it triggered calcium burst activities that rapidly altered the calcium concentration gradient, consequently unbalancing the process loading. It also activated calcium-dependent pathways in the model and increased the simulation time for reaction SSA operations.

**Figure 8:**
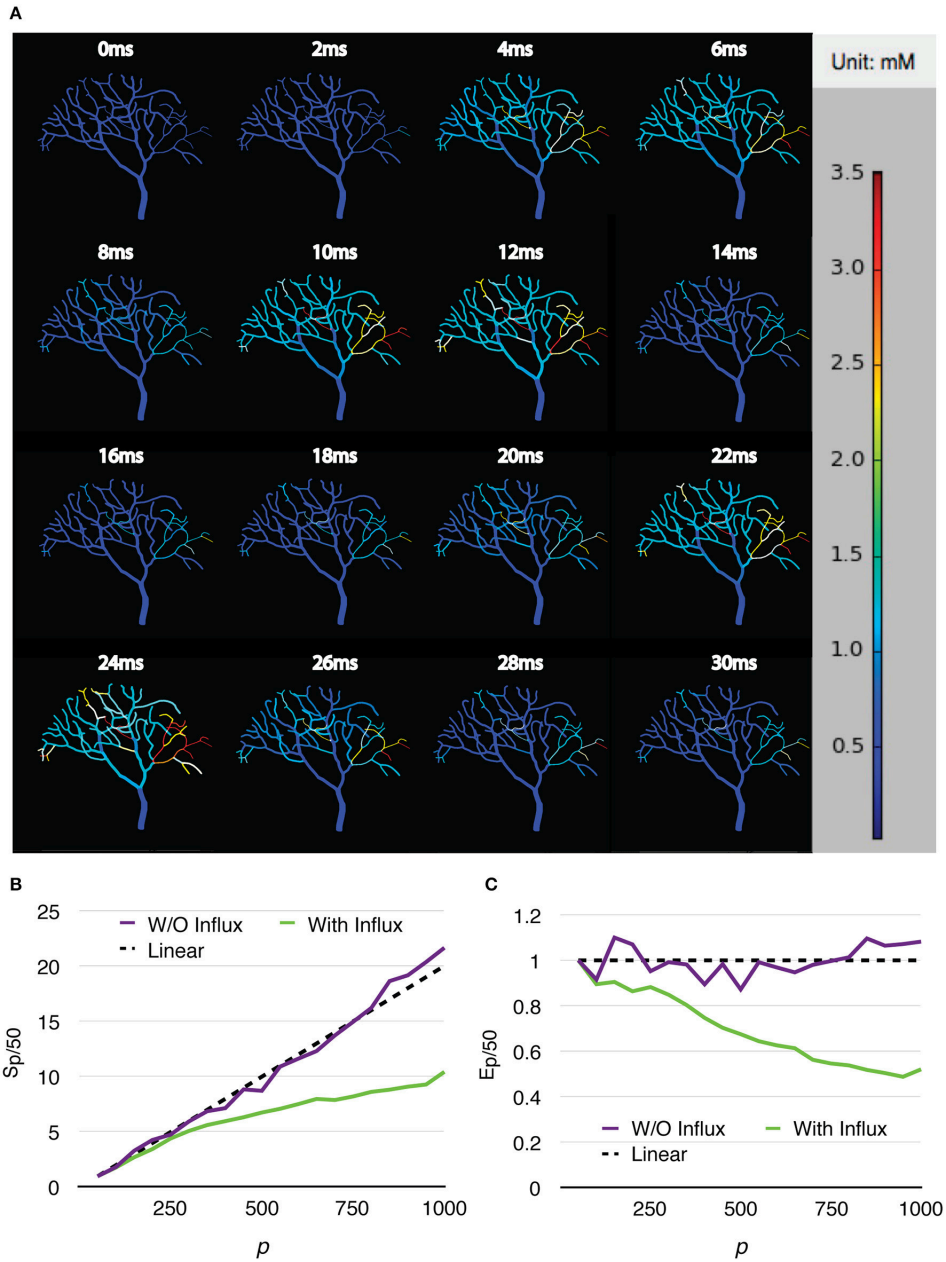
**Calcium burst simulations with a Purkinje cell sub-branch morphology. (A)** Calcium activity of each branch segment over a single trial period, visualized by the STEPS visualization toolkit. Calcium activity shows large spatial and temporal variability, which significantly affects the speedup **(B)** and efficiency **(C)** of the simulation.

**Figure 9:**
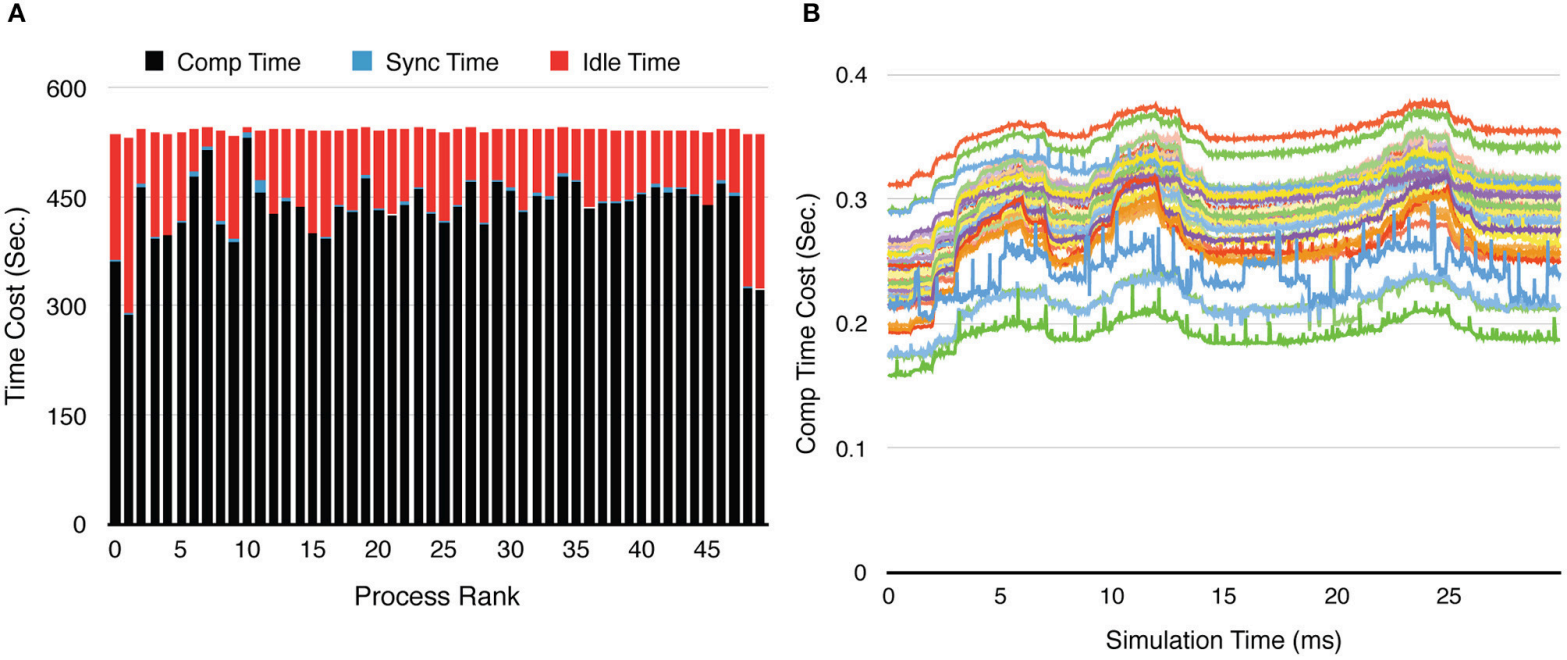
**Process loading of a calcium burst simulation with sub-branch morphology and calcium influx, using 50 processes. (A)** Time-cost distribution for each process shows the loading imbalance across processes. **(B)** The computation time cost per recording step for each process varies significantly during the simulation. Each curve in the figure represents one process. The three peaks in each curve are caused by the three burst periods (Figure 8A).

Two series were simulated, one without calcium influx and data recording, and the other one with the influx enabled and data recorded periodically. Each series of simulations started from *p* = 50, and finished at *p* = 1,000, with an increment of 50 processes each time. Both series of simulations were run for 30 ms, and repeated 20 times to acquire the average wall-clock times. For the simulations with calcium influx, the influx rate of each branch segment was adjusted according to the profile every 1 ms, and the calcium concentration of each branch was recorded to a file every 0.02 ms, as in the original simulation. Figure 8A shows the recorded calcium activity of each branch segment over a single simulation trial period, which exhibits great spatial and temporal variability as reported previously (Anwar et al., 2013). A video of the same simulation is also provided in the Supplementary Material (Video 1). As a consequence of calcium influx changes, process loading of the series was mostly unbalanced so that simulation speedup and efficiency were significantly affected. However, a substantial improvement was still achieved (Figures 8B,C). Figure 9 demonstrates the loading of an influx simulation with 50 processes, where the imbalance can be observed across processes and time. To improve the performance of simulations with strong concentration gradients, a sophisticated and efficient dynamic load balancing algorithm is required (see Discussion).

**Figure 10:**
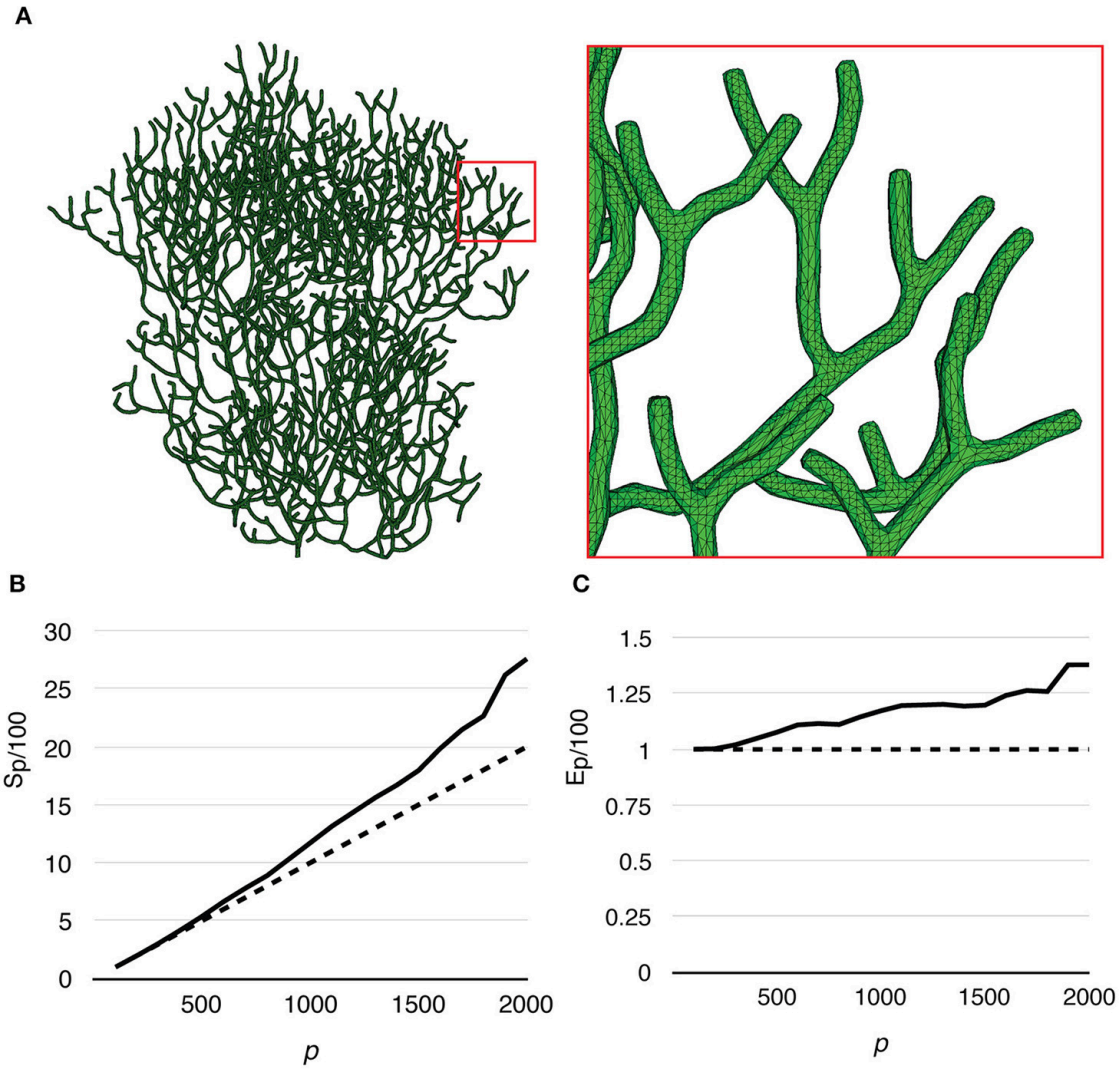
**Performance of a reaction-diffusion simulation with a mesh of a complete Purkinje dendrite tree. (A)** Morphology of the mesh and a close look at its branches. The mesh consists of 1,044,155 tetrahedrons. **(B)** Speedup relative to the simulation with *p* = 100 shows super-linear scalability. **(C)** Efficiency also increases as *p* increases, suggesting that better efficiency may be achieved with more processes.

Finally, to test the capability of our implementation for full cell stochastic spatial simulation in the future, we generated a mesh of a full Purkinje dendrite tree from a publically available surface reconstruction (3DModelDB; McDougal and Shepherd, 2015, ID: 156481) and applied the above model to it. To the best of our knowledge, this is the first parallel simulation of a mesoscopic level, stochastic, spatial reaction-diffusion system with full cell dendritic tree morphology. The mesh consisted of 1,044,155 tetrahedrons. Because branch diameters of the original reconstruction have been modified for 3D printing, the mesh is not suitable for actual biological study, but only to evaluate computational performance. Because of this, and the fact that no calcium influx profile can be acquired for this reconstruction, we only ran simulations without calcium influx. The simulation series started from *p* = 100, and progressively increased to *p* = 2000 by an increment of 100 processes each time. The maximum number of processes (*p* = 2000) was determined by the fair-sharing policy of the computing center. We repeated the series 20 times to produce the average result. Figure 10A gives an overview of the full cell morphology as well as a zoom-in look at the mesh. Both speedup and efficiency relative to simulation with *p* = 100 (Figures 10B,C) show super-linear scalability and has the best performance with *p* = 2000. This result suggests that simulation performance may be further improved with a higher number of processes.

**Figure 11:**
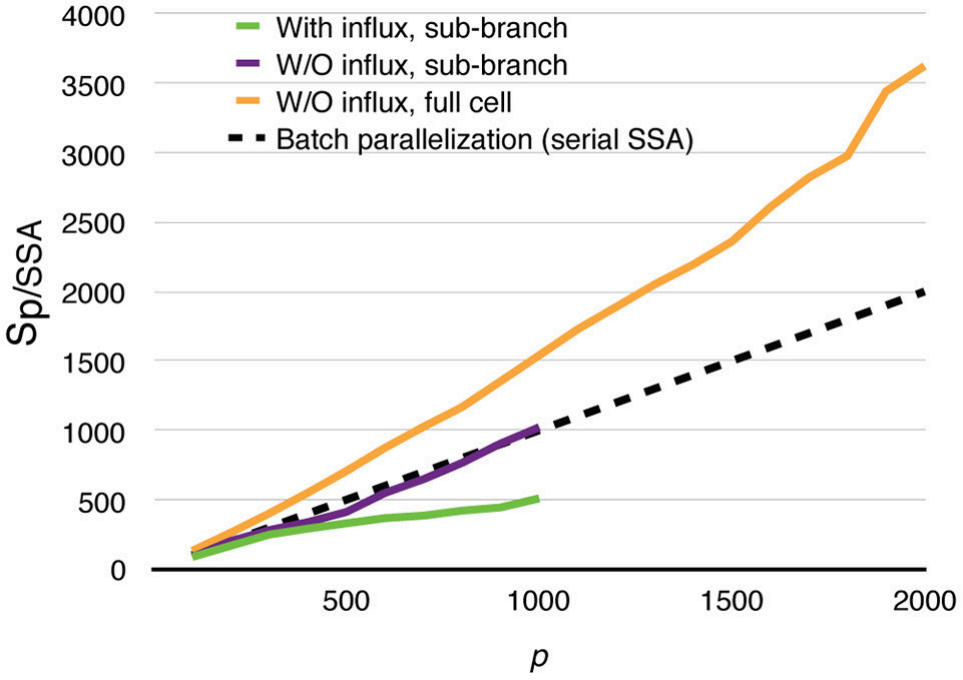
**Speedups of parallel calcium burst simulations relative to their serial SSA counterparts, including sub-branch simulations with and without calcium influx, and the full cell simulation without calcium influx**. The dashed curve assumes that *p* processes are used to simulate a batch of *p* serial SSA realizations of the full cell simulation.

All parallel simulations above perform drastically better than their serial SSA counterparts. For each of the test cases above, 20 realizations were simulated using the serial SSA solver in STEPS, and average wall-clock times are used for comparison. The speedups relative to the serial SSA simulations are shown in Figure 11. Even in the most realistic case, with dynamically updated calcium influx as well as data recording, without any special load balancing treatment, the parallel simulation with 1000 processes is still 500 times faster than the serial SSA simulation. The full cell parallel simulation without calcium influx achieves an unprecedented 3600-fold speedup with 2000 processes. This means with full usage of the same computing resources and time, parallel simulation is not only faster than single serial SSA simulation, but is also 1.8 times the speed of batch serial SSA simulations.

## Discussion and future directions

Our current parallel STEPS implementation achieves significant performance improvement and good scalability, as shown in our test results. However, as a preliminary implementation, it lacks or simplifies several functionalities that could be important for real-world simulations. These functionalities require further investigation and development in future generations of parallel STEPS.

Currently, STEPS models with membrane potential as well as voltage-dependent gating channels (Hepburn et al., 2013) cannot be efficiently simulated using the parallel solver because a scalable parallelization of the electric field (E-Field) sub-system is still under development. This is the main reason why we were unable to fully simulate the stochastic spatial calcium burst model with Purkinje sub-branch morphology in our example, but relied on the calcium influx profile extracted from a previous serial simulation instead. The combined simulation of neuronal electrophysiology and molecular reaction-diffusion has recently raised interest, as it bridges the gap between computational neuroscience and systems biology, and is expected to be greatly useful in the foreseeable future. To address such demand, we are actively collaborating with the Human Brain Project (Markram, 2012) on the development of a parallel E-Field, which will be integrated into parallel STEPS upon its completion.

As analyzed in the results, the majority of the performance speedup is contributed by the reduction of *T*_*comp*_, thanks to parallel computing. Eventually *T*_*idle*_ becomes the main bottleneck, as it is mostly constant relative to the process count, unlike *T*_*comp*_ which decreases consistently. This observation suggests two future investigational and developmental directions, maximizing the speedup gained from *T*_*comp*_, and minimizing *T*_*idle*_.

Maximizing the speedup gained from *T*_*comp*_ is important to real-world research because significant performance improvement needs to be achieved with reasonable computing resources. Adapting advanced algorithms and optimizing memory caching are two common approaches to achieve this goal. At present, we mainly focus on further optimizing memory footprint and caching ability for super-large scale simulations. In the current implementation, although reaction SSA and propensity update information are distributed, each process still stores complete information of the simulation state. This noticeably affects the weak scalability of our implementation (Figure 6). The redundant information is so far required for the purpose of interfacing with other non-parallel sub-systems, such as serial E-Field, but we will investigate whether state information can be split, based on the demand of individual processes.

Process load balancing plays a crucial role in determining the idle time of the simulation *T*_*idle*_, and consequently the maximum speed improvement the simulation can achieve. In an unbalanced-loading simulation, processes will always be idle until the slowest one finishes, thus dramatically increasing *T*_*idle*_. This issue is essential to spatial reaction-diffusion simulations as high concentration gradients of molecules can be observed in many real-world models, similar to our calcium burst model. Because molecule concentrations change significantly during simulation due to reactions and diffusion, the loading of each process may change rapidly. While adding model and initial molecule concentration information to the partitioning procedure may help to balance the loading for early simulation, the initial partitioning will eventually become inefficient as molecule concentrations change. An efficient load balancing algorithm is required to solve this problem. The solution should be able to redistribute tetrahedrons between processes automatically on the fly based on their current workloads. Data exchange efficiency is the main focus of the solution, because constantly copying tetrahedron data between processes via network communication can be extremely time consuming, and may overshadow any benefit gained from the rebalancing.

In its current status, our parallel STEPS implementation constitutes a great improvement over the serial SSA solution. The calcium burst simulation with Purkinje cell sub-branch morphology, dynamic calcium influx, and periodic data recording is representative of the simulation condition and requirements of typical real-world research. Similar models that previously required years of simulation can now be completed within days. The shortening of the simulation cycle is greatly beneficial to research as it provides opportunities to further improve the model based on simulation results.

## Code availability

Parallel STEPS can be accessed via the STEPS homepage (http://steps.sourceforge.net), or the HBP Collaboration Portal (https://collaboration.humanbrainproject.eu). Simulation scripts for this manuscript are available at ModelDB (https://senselab.med.yale.edu/modeldb/).

## Author contributions

WC designed, implemented and tested the parallel STEPS described, and drafted the manuscript. ED conceived and supervised the STEPS project and helped draft the manuscript. Both authors read and approved the submission.

### Conflict of interest statement

The authors declare that the research was conducted in the absence of any commercial or financial relationships that could be construed as a potential conflict of interest.

## Supplementary material

Video 1 **Data recording of a calcium burst simulation with Purkinje cell sub-branch morphology, visualized using STEPS visualization toolkit**.
