# Supplementary material for: Parallel STEPS: Large Scale Stochastic Spatial Reaction-Diffusion Simulation with High Performance Computers
Source: Front Neuroinform. 2017 Feb 10;11:13. doi: 10.3389/fninf.2017.00013 (PMC5301017; doi:10.3389/fninf.2017.00013)
Supplement: Supplementary file 2 [file DataSheet1.docx]

**Algorithms**

Algorithm 1: Parallel STEPS Runtime Main Loop

The runtime main loop is split into three major components, the Computation Period, including the Reaction SSA Operator and the Diffusion Operator, the Cross-Process Synchronization Period, as well as the Idle Period. The mean number of a molecular species *S* present in a tetrahedron *tet* during a diffusion period $\tau$, $\bar{N_{S}}$, is calculated by the molecule update time $t_{S}$, and the fractional molecule occupancy $O_{S}$ (Hepburn et al., 2016). It is then used to compute the number of *S* to be distributed among neighbors of *tet*. The solver waits for the communication of all buffers from the previous iteration to complete before executing the diffusion operator. While local diffusion events are applied directly, cross-process diffusion events are registered in remote change buffers and sent to corresponding processes for update.

Here we denote $p$ as the running process, *neighs_p* as the set of processes hosting tetrahedrons that are neighbors to any tetrahedron hosted by $p$, and $Reacs$ as the set of all reactions in tetrahedrons hosted by $p$, on which the SSA kernel is operated.

1. **for** each $p^{'}$ in *neighs_p* **do**
2. **create** $remote\_change\_buffer\left[ p^{'} \right]$
3. **end for**
4. **initialize** buffer_exchange_in_progress = False
5. **while** $t<t_{end}$ **do**
6. **if** $t+\tau>t_{end}$ **then**
7. $\tau=t_{end}-t$
8. **end if**

# Reaction SSA Operator

1. **initialize** $\Delta t=0.0$
2. **for** each reactant and product *S* in each reaction *R* in $Reacs$ **do**
3. **initialize** $t_{S}=0.0$
4. **initialize** $O_{S}=0.0$
5. **end for**
6. **while** $\Delta t\leq\tau$ **do**
7. compute the next reaction time $t_{next}$ using SSA
8. **if** $\Delta t+t_{next}>\tau$ **then** **break**
9. **end if**
10. find the next reaction event $R_{next}$ using SSA
11. **if** no $R_{next}$ is found **then break**
12. **end if**
13. $\Delta t+=t_{next}$
14. apply molecule changes caused by $R_{next}$
15. **for** each reactant and product species *S* of $R_{next}$ **do**
16. $O_{S}+=N_{S}(\Delta t-t_{S})$, where $N_{S}$ is the previous molecule count of *S* at $t_{S}$
17. $t_{S}=\Delta t$
18. **end for**
19. **end while**

# Idle Period

1. **if** buffer_exchange_in_progress == True **then**
2. wait for all buffers to be sent
3. buffer_exchange_in_progress = False
4. **end if**
5. **for** each $p^{'}$ in *neighs_p* **do**
6. **reset** $remote\_change\_buffer\left[ p^{'} \right]$
7. **end for**

# Diffusion Operator

1. **for** each diffusive species *S* in *tet* hosted by *p* **do**
2. $O_{S}+=N_{S}(\tau-\Delta t)$
3. $\bar{N_{S}}=O_{S}/ \tau$
4. distribute *n* molecules among neighbors of *tet*, where $n=binomial(\bar{N},\tau,d_{S, tet})$
5. **for** each neighboring tetrahedron, *neigh_tet* **do**
6. **if** *neigh_tet* is hosted by *p* **then** #local diffusion

apply molecule changes for tet and *neigh_tet*

1. **else if** *neigh_tet* is hosted by $p'$, $p'\neq p$ **then** #cross-process diffusion
2. apply molecule changes for *tet*
3. register molecule changes for *neigh_tet* to $remote\_change\_buffer\left[ p^{'} \right]$ # Algorithm 2
4. **end if**
5. **end for**
6. **end for**
7. update affected propensities of hosted reactions and diffusions in *p*

# Cross-Process Synchronization Period

1. **for** each $p^{'}$ in *neighs_p* **do**
2. send $remote\_change\_buffer\left[ p^{'} \right]$ to $p^{'}$ using non-blocking communication
3. **end for**
4. buffer_exchange_in_progess = True
5. **initialize** n_buffer_received = 0
6. **while** n_buffer_received ≠ sizeof(*neighs_p*) **do** # wait and receive buffers then apply them
7. wait until a buffer arrives
8. receive buffer
9. apply molecule changes and update propensities according to the buffer data
10. n_buffer_received += 1
11. **end while**
12. $t += \tau$
13. **end while**

**(cont.)**

**(Algorithm 1 cont.)**

# Idle Period

1. **if** buffer_exchange_in_progress == True **then**
2. wait for all buffers to be sent
3. buffer_exchange_in_progress = False
4. **end if**
5. **for** each $p^{'}$in *neighs_p* **do**
6. **delete** $remote\_change\_buffer\left[ p^{'} \right]$
7. **end for**

Algorithm 2: Registration of a cross-process diffusion event

This pseudocode illustrates the registration procedure of a cross-process diffusion event $D_{tet\to{tet}^{'}, S}$ from tetrahedron $tet$ in process $p$ to neighboring tetrahedron ${tet}^{'}$ in process $p^{'}$with diffusive species *S*. For simplification, $remote\_change\_buffer\left[ p^{'} \right]$ is denoted as *buffer* here, which has the same structure as in Figure 2. We also denote $n_{{tet}^{'}}$ as the amount of molecule *S* in ${tet}^{'}$ changed by $D_{tet\to{tet}^{'}, S}$ each time. Note that $n_{{tet}^{'}}$ can be more than 1 due to the use of the binomial distribution in Algorithm 1.

1. **retrieve** ${Loc}_{{tet}^{'},S}$ from $D_{tet\to{tet}^{'}, S}$ stored in $tet$
2. **if** sizeof(*buffer*) > ${Loc}_{{tet}^{'},S}$ and *buffer*[${Loc}_{{tet}^{'},S}$] == ${tet}^{'}$ and *buffer*[${Loc}_{{tet}^{'},S}+1$] == *S* **then**
3. *buffer*[${Loc}_{{tet}^{'},S}+2$] += $n_{{tet}^{'}}$
4. **else**
5. ${Loc}_{{tet}^{'},S}$ = sizeof(*buffer*)
6. **append** ${tet}^{'}$, *S*, $n_{{tet}^{'}}$ to the end of *buffer*
7. **end if**
